# Complete Chloroplast Genome of Medicinal Plant *Lonicera japonica*: Genome Rearrangement, Intron Gain and Loss, and Implications for Phylogenetic Studies

**DOI:** 10.3390/molecules22020249

**Published:** 2017-02-07

**Authors:** Liu He, Jun Qian, Xiwen Li, Zhiying Sun, Xiaolan Xu, Shilin Chen

**Affiliations:** 1 Institute of Medicinal Plant Development, Chinese Academy of Medical Sciences & Peking Union Medical College, Beijing 100193, China; lhe@implad.ac.cn (L.H.); jqian@implad.ac.cn (J.Q.); zysun@implad.ac.cn (Z.S.); xlxu@implad.ac.cn (X.X.); 2 Institute of Chinese Materia Medica, Academy of Chinese Medical Sciences, Beijing 100700, China

**Keywords:** *Lonicera japonica*, chloroplast genome, contraction

## Abstract

The complete chloroplast (cp) genome of *Lonicera japonica*, a common ornamental and medicinal plant in North America and East Asia, was sequenced and analyzed. The length of the *L. japonica* cp genome is 155,078 bp, contains a pair of inverted repeat regions (IRa and IRb), of 23,774 bp each, as well as large (LSC, 88,858 bp) and small (SSC, 18,672 bp) single-copy regions. A total of 129 genes were identified in the cp genome, 16 of which were duplicated within the IR regions. Relative to other plant cp genomes, the *L. japonica* cp genome had a unique rearrangement between trnI-CAU and trnN-GUU. In *L. japonica* cpDNA, *rps19*, *rpl2*, and *rpl23* move to the LSC region, from the IR region. The *ycf1* pesudogene in the IR region is lost, and only one copy locates in the SSC region. Comparative cp DNA sequence analyses of *L. japonica* with other cp genomes reveal that the gene order, and the gene and intron contents, are slightly different. The introns in *ycf2* and *rps18* genes are found for the first time. Four genes (*clpP*, *petB*, *petD*, and *rpl16*) lost introns. However, its genome structure, GC content, and codon usage were similar to those of typical angiosperm cp genomes. All preferred synonymous codons were found to use codons ending with A/T. The AT-rich sequences were less abundant in the coding regions than in the non-coding ones. A phylogenetic analysis based on 71 protein-coding genes supported the idea that *L. japonica* is a sister of the Araliaceae species. This study identified unique characteristics of the *L. japonica* cp genome that contribute to our understanding of the cpDNA evolution. It offers valuable information for the phylogenetic and specific barcoding of this medicinal plant.

## 1. Introduction

*L. japonica* is a sprawling and twining liana of the genus *Lonicera* in Caprifoliaceae and Dipsacales. It is native to eastern Asia and was cultivated as medicinal plant with great economic value. The *Lonicera* genus has almost 100 species in China, and half of them have medicinal effects, including *L. japonica*, *L. macranthoides* [1], *L. similis* [2], *L. fulvotomentosa* [3], and *L. hypoglauca* [4]. To date, more than 140 compounds have been isolated and identified from *L. japonica* [5]. The dried flowers, buds, and leaves of *L. japonica* are widely used with other Chinese medicines in the treatment of epidemic febrile and infectious diseases, such as SARS and avian influenza [6].

Centuries ago, *L. japonica* was introduced to North America, South America, and Oceania as an ornamental plant [7]. Now, it is well-known in America as a horticultural plant with wind breaker and sand-fixation properties. *L. japonica* does not wither, even in winter, where mean temperatures are at least −1 °C, and is very effective for ecological protection in China.

However, *L. japonica* is the only species within the genus used as traditional Chinese Medicine, and species identification of *L. japonica*, from other *Lonicera* species, is quite difficult. Molecular barcodes based on the cp genome have shown great potential for species discrimination, especially between closely related taxa [8]. The complete chloroplast genome sequence might enhance our ability to explore reliable barcoding for accurate plant identification, at both the species and population levels [9]. 

In higher plants, photosynthesis occurs in the cp, to provide the essential energy needed for plant growth and survival. New leaves of *L. japonica* have higher photosynthetic rates than other *Lonicera* species, whether they are under the forest canopy or in the open [7]. The annual carbon gain for Japanese honeysuckle was much greater in different light environments [10]. However, the molecular mechanism of photosynthetic adaptability of *L. japonica* is still beyond our outstanding. The lack of the cp genome of *L. japonica* has become a bottleneck for investigating whether there are links between *L. japonica*’s high level adaptability and photosynthetic adaptability, as well as chloroplast function. With rapid advances in sequencing technologies, Herbgenomics provides an effective tool to uncover the genetic information of herbs and to clarify their molecular mechanisms in related biological responses [11,12].

Although the transcriptome sequences of *L. japonica* have been previously reported [13], this study is the first to report its cp genome sequence. Comparative analyses among cp genomes of Apiales species revealed changes in the genome sizes, as well as the loss of genes and introns. Our data will help to identify the genetic and evolutionary mechanisms required for an in-depth study of *L. japonica*, and will be beneficial for DNA barcoding studies in *Lonicera*.

## 2. Results and Discussion

### 2.1. Characteristics of L. japonica cpDNA

**Figure 1:**
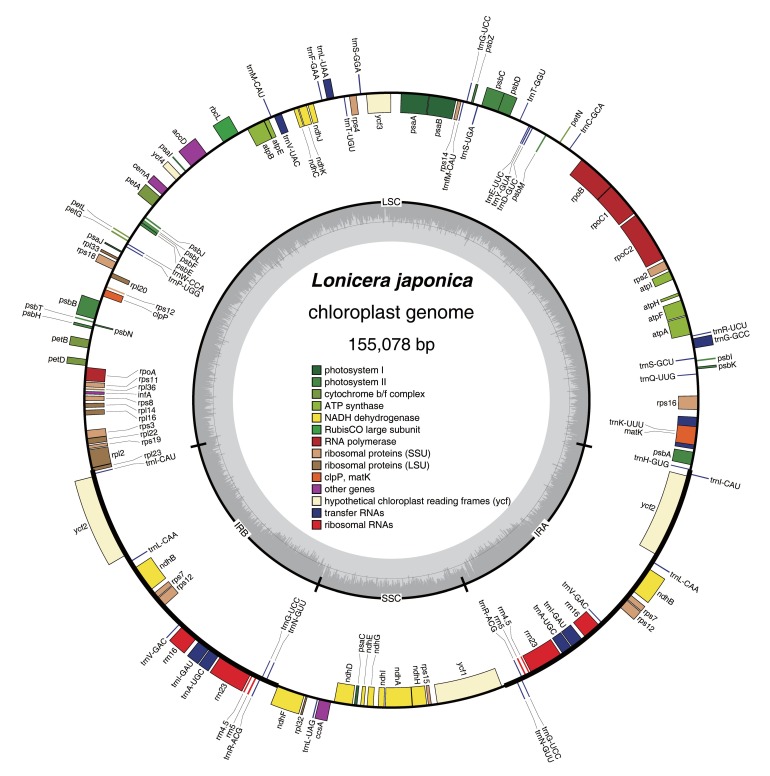
Map of the *L. japonica* chloroplast genome. Genes drawn inside the circle are transcribed in a clockwise direction, whereas those outside the circle are transcribed in a counterclockwise direction. Genes belonging to the same functional groups have the same colors.

The library was constructed from the cpDNA of *L. japonica* leaves with the 454 GS FLX Titanium platform, using the manufacturer’s manual. A total of 22,185 reads were obtained, with an average length of 412 bp, yielding approximately 58× coverage of the cp genome. The complete cp genome of *L. japonica* is 155,078 bp in length (Accession No. KJ170923). Its genome exhibits a typical quadripartite structure that consists of a pair of IR regions (23,774 bp), separated by the LSC (88,858 bp) and SSC (18,672 bp) regions (Table 1, Figure 1).

A total of 79 protein-coding genes, 30 tRNA genes, and 4 rRNA genes, were annotated (Table S1). These genes have been retained in several angiosperms [14,15,16,17,18]. Among these genes, eight tRNA genes, four rRNA genes, and four protein-coding genes, were duplicated in the IR regions. The LSC region contains 63 protein-coding and 22 tRNA genes, whereas the SSC region contains one tRNA gene and 12 protein-coding genes.

The majority (52%) of the *L. japonica* cp genome is composed of non-coding regions, including introns, intergenic spacers, and pseudogenes. The overall GC and AT content of the *L. japonica* cp genome was 38.6% and 61.4%, respectively. The AT content of the LSC, SSC, and IR regions was 62.9%, 66.6% and 56.5%, respectively (Table 1). Within the protein-coding regions (CDS), the AT content of the first, second, and third codon positions, is 53.7%, 61.6% and 68.8%, respectively (Table 1). The bias toward a higher AT representation at the third codon position is generally found in plant cp genomes; this bias is used to distinguish cpDNA from nuclear DNA and mitochondrial DNA [14,15,16,19]. Based on the sequences of the protein-coding and tRNA genes, the frequency of codon usage was deduced for the *L. japonica* cp genome and summarized in Table 2. The high AT content at the third codon position reflects a codon usage bias for A or T. The codon usage frequencies of stop codons are similarly biased to A or T at the second and third codon positions. A total of 2692 codons (10.6%) encoded for leucine, whereas 273 (1.1%) encoded for cysteine, which are the most and least prevalent amino acids, respectively. A general excess of A- and U-ending codons was noted. Except for trL-CAA, all of the types of preferred synonymous codons (RSCU > 1) ended with A or U (Table 2).

### 2.2. Intron Gain and Loss

Advances in phylogenetic research have demonstrated that cp genome evolution includes nucleotide substitutions and structural changes [20,21]. A few examples of these changes, including gene or intron losses, have been found in cp genomes [22,23,24,25,26,27]. Previous study has demonstrated that introns had important roles in alternative splicing, and it has been demonstrated that the introns can significantly stabilize the transcripts in some eukaryotic lineages [28]. Additionally, orthologous genes are also believed to have lost or gained introns throughout evolution. To provide more information for further study on cp genome evolution in *Caprifoliaceae* and the potential functional change from variations of intron gain and loss, we analyzed the cp genome of *L. japonica*.

In total, there are 16 intron-containing genes, 14 of which contain one intron, and two of which (*rps18* and *ycf3*) contain two introns (Table 3). The ribosomal protein S18 is essential for plastid translation in plant development [29]. Ycf3 is required for the stable accumulation of the photosystem I complex. In green alga *Chlamydomonas reinhardtii*, *ycf3* and *rps18* genes belong to the *rps9-ycf4-ycf3-rps18* polycistronic transcriptional unit. In land plants, *ycf3* and *rps18* are found in different clusters [30]. The presence of two introns in the *rps18* gene in the *L. japonica* cp genome, is rare. Similarly, the intron in *ycf3* was not previously mentioned in other cp genomes. The intron gain in several *L. japonica* cp genes is first reported, and the intron gain in *rps18* and *ycf3* of *L. japonica* may be useful for further studies on the mechanism of photosynthesis evolution. 

Compared to other cp genes, the introns in the *clpP*, *petB*, *petD*, and *rpl16* genes, were lost in the *L. japonica* cp genome. The *rpl16* intron is a highly stable component of angiosperm cp genomes; this intron is absent from very few taxa, namely the Geraniaceae, Goodeniaceae, and Plumbaginaceae families [31]. Similarly, previous studies have shown that introns are also absent in the *clpP* gene of the *Jasminum nudiflorum* cp genome [22,31]. The intron loss of *petB*, *petD,* and *rpl16* was first found in the lineages of Asterids. Introns are important in the regulation of gene expression. They can enhance the gene expression level, on the special position, in the specific time [16]. Some introns are known to enhance, or are required for, normal levels of mRNA transcription, processing, and transport. Several unicellular eukaryotes appear to be under selection pressure to lose introns. However, no studies between intron loss and gene expression, using transcriptome data from *L. japonica*, have been published. 

The intron density in eukaryote genomes varies by more than three orders of magnitude. Therefore, extensive intron gain and/or intron loss must have occurred during evolution. A common partial explanation for the range of intron densities, is the stochastic accumulation of introns in large eukaryote genomes during their evolution from an intron-poor ancestor. We still need more experimental information to reveal whether the variation of the introns in the *L. japonica* cp genome is related to the adaptability to stress*.*

### 2.3. Comparison with Other cp Genomes in the Order Apiales

**Figure 2:**
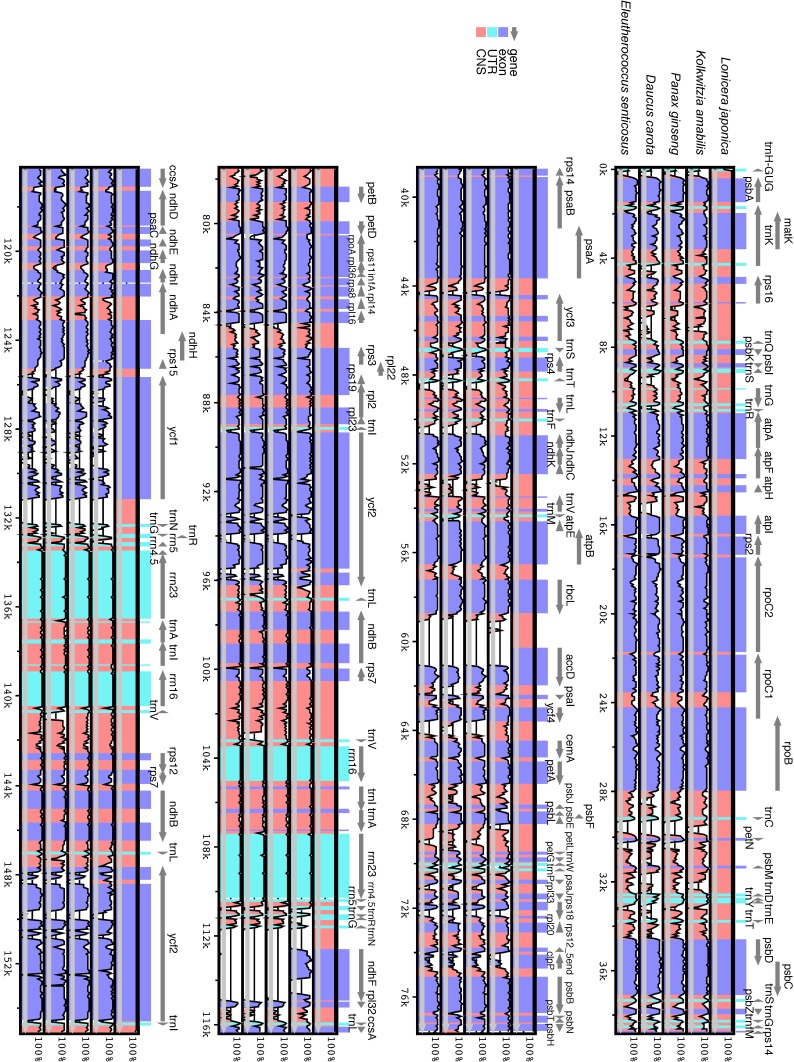
Comparison of four chloroplast genomes using mVISTA program. Grey arrows and thick black lines above the alignment indicate the orientation of genes and the position of the IR regions, respectively. Purple bars represent exons, blue ones represent UTRs, and pink ones represent non-coding sequences (CNS). A cut-off of 70% identity was used for the plots. The Y-scale axis represents the percent identity within 50%–100%. Genome regions are color-coded as either protein-coding exons, rRNAs, tRNAs, or conserved noncoding sequences (CNS).

Both *L. japonica* and *Kolkwitzia amabilis* [32] belong to the order Dipsacales (Figure S1). Apiales and Dipsacales are both Asterids. Some cp genomes in the Apiales clade have been reported, such as those of the *Eleutherococcus senticosus* [16], *Daucus carota* [17], and *Panax ginseng* [18] chloroplast. These representative cp genome sequences of Apiales were selected for comparison with those of *L. japonica* and *K. amabilis.* The overall sequence identification of the cp genomes was plotted using mVISTA, with the annotation of *L. japonica* as a reference (Figure 2). The length of the LSC and IR regions was the main difference between genomes (Table S2). The comparison showed that the two IR regions were less divergent than the LSC and SSC regions. Coding and non-coding regions were present, and the most divergent regions among the four cp genomes were localized to the intergenic spacers. These highly divergent regions were included in the alignment.

### 2.4. IR Contraction in the L. japonica cp Genome

**Figure 3:**
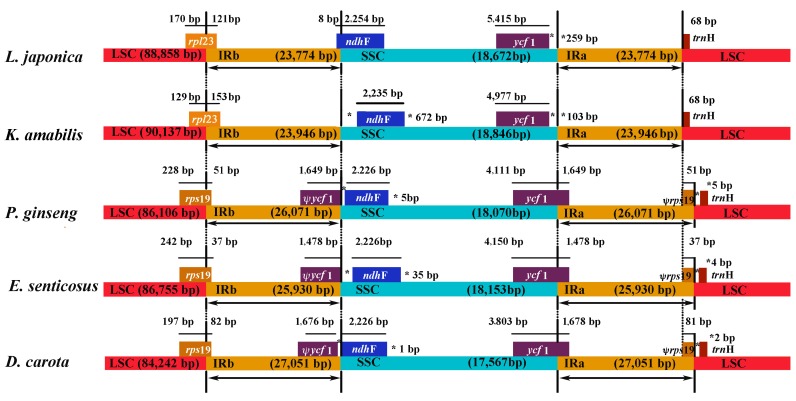
Comparison of the borders of the LSC, SSC, and IR regions among five chloroplast genomes. *Ψ*: pseudogenes, *: the distance from the edge.

The contraction and expansion at the borders of the IR regions are common evolutionary events and represent the main reasons for the size variation of cp genomes [33]. The ends of the IRa and IRb regions, as well as the gene length, differ among plant lineages. Detailed comparisons of IR-SSC and IR-LSC boundaries among the five Asterid cp genomes, are presented in Figure 3. Similar to the *N. tabacum* [34] and *Penthorum chinense* [35], the *rps19* gene existed in the LSC region. However, some unique structural differences exist: the contraction of the inverted repeat region, to exclude the *rpl2*, *rpl23*, *and ycf1* genes, typically exists in the IR region in other angiosperm cp genomes; however, they were not excluded from the LSC region in *L. japonica*. Moreover, *rps19*, *rpl2*, *rpl23*, and *ycf1* pseudogenes were found in *L. japonica*. In Spirogyra maxima, *trnI-rpl23-rpl2-rps19*- is a large operon of angiosperm chloroplast genomes. The *rpl23* gene cluster of Spirogyra contains a distinct eubacterial promoter sequence, upstream of *rpl23*, which is the first gene of the green algal *rpl23* gene cluster. This sequence is completely absent in angiosperms, but is present in non-flowering plants. The results imply that, in the *rpl23* gene cluster, early charophytes had at least two promoters, one which was upstream of *trnI*, and another which was upstream of *rpl23*, which partially or completely lost its function in land plants [36]. The IRb/SSC border is generally located between the *ycf1* pseudogene and the *ndhF* gene. However, the *ycf1* pseudogene was absent in *L. japonica*. *Ycf1* pseudogenes have been proved useful for analyzing cp genome variation in higher plants and algae, even though their function is not thoroughly known. *Ycf1* and *ycf2* are essential for plant survival [37]. A combined analysis of the chloroplast genome and transcriptome of *Deschampsia antarctica Desv*, indicated that the *rps19* gene was one of the most abundant transcripts in the chloroplast’s genome [38]. The portion of the *ndhF* gene located in the IRb region was 8 bp long. The *rps19* and *ycf1* genes were not found in the IR region, and the two pseudogenes were absent in the cp genome. These features are reported for Asterid plants for the first time.

The cp genome size displayed among the examined Apiales species was compared. The length of the IR (23,774 bp) in *L. japonica* was 3278 bp smaller than that of *D. carota*, 2157 bp smaller than that of *E. senticosus*, and 2298 bp smaller than that of *P. ginseng.* These differences could be attributed to the loss of the *rpl2* and *ycf15* genes, as well as the *ycf1* and *rps19* pseudogenes in *L. japonica* IR regions. However, the length of the whole genome was not significantly different among the five Asterid cp genomes. The genome of *L. japonica* (155,078 bp) was 834 bp smaller than that of *D. carota*, 1691 bp smaller than that of *E. senticosus*, and 1241 bp smaller than that of *P. ginseng.* Non-functional DNA was likewise rapidly deleted, resulting in the failure of pseudogenes to accumulate, despite the high rates of pseudogenes.

### 2.5. Phylogenetic Analysis

**Figure 4:**
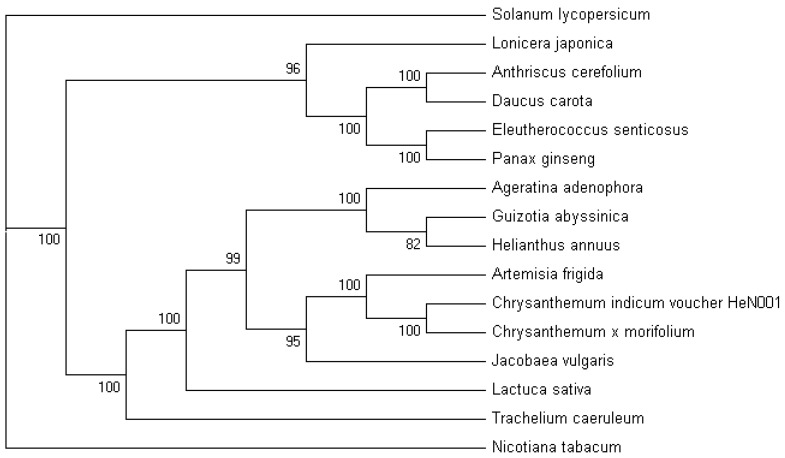
MP phylogenetic tree of the Apiales clade based on 71 protein-coding genes. The MP tree has a length of 17,973, with a consistency index of 0.8080 and a retention index of 0.8285. Numbers above each node are the bootstrap support values. *Solanum lycopersicum* and *Nicotiana tabacum* were set as the outgroups.

The gene content of cpDNA is highly conserved among most land plants. The cp genome sequence is a useful resource for studying the taxonomic status of the genus *Lonicera* in the angiosperm clade, and for analyzing evolutionary relationships within the family [22]. To obtain a reasonable phylogenetic status of *Lonicera*, we performed multiple sequence alignments of protein coding genes, from a variety of plant plastomes. A total of 15 complete cp genomes represented six families, within five orders. Phylogenetic analysis was performed on a 71-gene data matrix, using MP and ML methods. MP analysis resulted in a single tree with a length of 17,973, a consistency index (CI) of 0.8080, and a retention index (RI) of 0.8285 (Figure 4). Bootstrap analysis showed that 12 out of the 13 nodes had bootstrap values >95%.

## 3. Materials and Methods

### 3.1. DNA Sequencing, Genome Assembly, and Validation

Fresh *L. japonica* leaves were collected from cultivated fields in Zhengcheng, Shandong Province, China. The samples used in this study were obtained from a local company (Jintai Yaoye Co., Ltd., Linyi, China). The total chloroplast DNA (cpDNA) was extracted from approximately 100 g of leaves via a sucrose gradient centrifugation method that was improved by Li et al. [39]. The cpDNA concentration for each sample was estimated by measuring *A*_260_ with an ND-2000 spectrometer (Nanodrop Technologies, Wilmington, DE, USA), whereas visual approximation was performed using gel electrophoresis. Pure cpDNA was used to construct shotgun libraries with the 454 GS FLX Titanium platform, according to the manufacturer’s instructions. This results in approximately 58× coverage of the cp genome. The obtained Sff-file was pre-processed, including the trimming of low-quality (Q < 20) and short (L < 50 bp) reads. The trimmed and cleaned reads were used for sequence assembly with the GS FLX De Novo Assembler Software (Newbler V2.6). To verify the assembly, four junction regions between the IR regions and LSC/SSC were confirmed by PCR amplifications and Sanger sequencing, with the primers listed in Table S3. The final cp genome sequence of *L. japonica* was then submitted to GenBank (Accession Number: KJ170923).

### 3.2. Gene Annotation and Sequence Analyses

Gene annotation was performed using BLAST and DOGMA [40]. The tRNA genes were identified using DOGMA and tRNAscanSE [41]. The circular cp genome map was drawn using the OGDRAW program [42]. To analyze the characteristics of the variations in synonymous codon usage, by neglecting the influence of amino acid composition, the relative synonymous codon usage values (RSCU), codon usage, and AT content, were determined using MEGA5.2 [43].

### 3.3. Genome Comparison

MUMmer [44] was used to perform pairwise cp genomic alignment. The mVISTA [45] program in the Shuffle-LAGAN mode [46], was used to compare the cp genome of *L. japonica* with the cp genomes of *K. amabilis*, *P. ginseng*, *D. carota*, and *E. senticosus* (KT966716, AY582139, DQ898156 and NC_016430), with the annotation of *L. japonica* as the reference. REPuter [47] was used to visualize the forward and inverted repeats.

### 3.4. Phylogenetic Analysis

A total of 15 complete cp genome sequences were downloaded from the NCBI Organelle Genome Resources database. For the phylogenetic analysis, a set of 71 protein-coding genes that were common in the 16 analyzed genomes, was used. Maximum parsimony (MP) analysis was performed with PAUP*4.0b10 [48], using a heuristic search combined with the random addition of 1000 replicates and tree bisection-reconnection (TBR) branch swapping, in the Multrees option. Bootstrap analysis was also performed with 1000 replicates and TBR branch swapping. *Solanum lycopersicum* and *Nicotiana tabacum* were set as outgroups.

## 4. Conclusions

High-throughput pyrosequencing technology was used to describe the completely sequenced *L. japonica* cp genome, which is a very important medicinal plant in East Asia. Compared to the cp genomes of three Apiales species, the cp genome of *L. japonica* has a relatively small size. Several genes were absent in the IR region, including the *rps19*, *rpl2*, and *ycf1* pseudogenes. This absence may be attributed to the obvious contraction of the IR region in *L. japonica*. Phylogenetic relationships among 15 angiosperms strongly supported the known classification of *L. japonica*. The data presented in this study can facilitate the biological identification of this important medicinal plant. Our data reveal that *L. japonica* cpDNA possesses several unique features that contribute to our current understanding of cpDNA evolution in seed plants. Additionally, other Dipsacales plastomes need to be sequenced to determine whether the atypical characteristics of *L. japonica* cpDNA are shared by all Dipsacales species, or if these characteristics are the unusual genomic features of a very unique plant.

## Figures and Tables

**Table 1:** Base composition in the *L. japonica* chloroplast genome.

		T(U) (%)	C (%)	A (%)	G (%)	Length (bp)
LSC		32.1	19.0	30.8	18.1	88,858
SSC		34.3	16.9	32.3	16.5	18,672
IRa		28.6	23.1	27.9	20.4	23,774
IRb		27.9	20.4	28.6	23.1	23,774
Total		31.2	19.6	30.2	19.0	155,078
CDS		31.3	18.1	30.0	20.6	74,724
	1st position	23.7	19.2	30.0	27.1	24,908
	2nd position	32.8	20.5	28.8	17.9	24,908
	3rd position	37.5	14.4	31.3	16.8	24,908

CDS: protein-coding regions.

**Table 2:** Codon–anticodon recognition patterns and codon usage of the *L. japonica* chloroplast genome.

Amino Acid	Codon	No.	RSCU	tRNA	Amino Acid	Codon	No.	RSCU	tRNA
Phe	UUU	911	1.27		Tyr	UAU	720	1.6	
Phe	UUC	527	0.73	*trnF-GAA*	Tyr	UAC	182	0.4	*trnY-GUA*
Leu	UUA	785	1.75	*trnL-UAA*	Stop	UAA	44	1.63	
Leu	UUG	570	1.27	*trnL-CAA*	Stop	UAG	21	0.78	
Leu	CUU	589	1.31		His	CAU	448	1.54	
Leu	CUC	202	0.45		His	CAC	135	0.46	*trnH-GUG*
Leu	CUA	377	0.84	*trnL-UAG*	Gln	CAA	666	1.5	*trnQ-UUG*
Leu	CUG	169	0.38		Gln	CAG	220	0.5	
Ile	AUU	972	1.42		Asn	AAU	857	1.5	
Ile	AUC	418	0.61	*trnI-GAU*	Asn	AAC	284	0.5	*trnN-GUU*
Ile	AUA	660	0.97	*trnI-CAU*	Lys	AAA	942	1.43	*trnK-UUU*
Met	AUG	582	1	*trn(f)M-CAU*	Lys	AAG	371	0.57	
Val	GUU	521	1.5		Asp	GAU	827	1.6	
Val	GUC	172	0.5	*trnV-GAC*	Asp	GAC	205	0.4	*trnD-GUC*
Val	GUA	490	1.41	*trnV-UAC*	Glu	GAA	902	1.44	*trnE-UUC*
Val	GUG	204	0.59		Glu	GAG	354	0.56	
Ser	UCU	554	1.75		Cys	UGU	191	1.4	
Ser	UCC	312	0.99	*trnS-GGA*	Cys	UGC	82	0.6	*trnC-GCA*
Ser	UCA	366	1.16	*trnS-UGA*	Stop	UGA	16	0.59	
Ser	UCG	171	0.54		Trp	UGG	454	1	*trnW-CCA*
Pro	CCU	402	1.47		Arg	CGU	318	1.26	*trnR-ACG*
Pro	CCC	209	0.77		Arg	CGC	95	0.38	
Pro	CCA	330	1.21	*trnP-UGG*	Arg	CGA	367	1.45	
Pro	CCG	150	0.55		Arg	CGG	113	0.45	
Thr	ACU	515	1.65		Arg	AGA	377	1.19	*trnR-UCU*
Thr	ACC	226	0.72	*trnT-GGU*	Arg	AGG	117	0.37	
Thr	ACA	369	1.18	*trnT-UGU*	Ser	AGU	456	1.8	
Thr	ACG	140	0.45		Ser	AGC	168	0.66	*trnS-GCU*
Ala	GCU	603	1.76		Gly	GGU	544	1.27	
Ala	GCC	227	0.66		Gly	GGC	189	0.44	*trnG-GCC*
Ala	GCA	388	1.13	*trnA-UGC*	Gly	GGA	646	1.51	*trnG-UCC*
Ala	GCG	155	0.45		Gly	GGG	331	0.77	

RSCU: Relative Synonymous Codon Usage.

**Table 3:** Genes with introns in the *L. japonica* chloroplast genome, including the exon and intron length.

Gene	Location	Exon I (bp)	Intron I (bp)	Exon II (bp)	Intron II (bp)	Exon III (bp)
*atpF*	LSC	411	735	144		
*ndhA*	SSC	539	1093	553		
*ndhB*	IR	756	679	777		
*rpl2*	IR	434	661	394		
*ycf2*	IR	498	249	6105		
*rpoC1*	LSC	430	775	1619		
rps12 *	LSC	114	-	232	536	26
*rps16*	LSC	230	869	40		
*rps18*	LSC	39	63	202	309	47
*trnA-UGC*	IR	35	809	38		
*trnG-UCC*	LSC	47	717	23		
*trnI-GAU*	IR	35	947	37		
*trnK-UUU*	LSC	35	2535	37		
*trnL-UAA*	LSC	49	515	36		
*trnV-UAC*	LSC	36	565	40		
*ycf3*	LSC	155	758	226	732	126

* The *rps12* gene is divided into 5′-rps12 in the LSC region and 3′-rps12 in the IR region.

## Supplementary Materials

Supplementary materials can be accessed at: http://www.mdpi.com/1420-3049/22/2/249/s1.
