# Supplementary material for: Complete Chloroplast Genome of Medicinal Plant Lonicera japonica: Genome Rearrangement, Intron Gain and Loss, and Implications for Phylogenetic Studies
Source: Molecules. 2017 Feb 7;22(2):249. doi: 10.3390/molecules22020249 (PMC6155926; doi:10.3390/molecules22020249)
Supplement: Supplementary file 1 [file molecules-22-00249-s001.pdf]

Supplementary Materials: Complete Chloroplast Genome of Medicinal Plant *Lonicera japonica*: Genome Rearrangement, Intron Gain and Loss and Implications for Phylogenetic Studies

Liu He, Jun Qian, Xiwen Li, Zhiying Sun, Xiaolan Xu and Shilin Chen

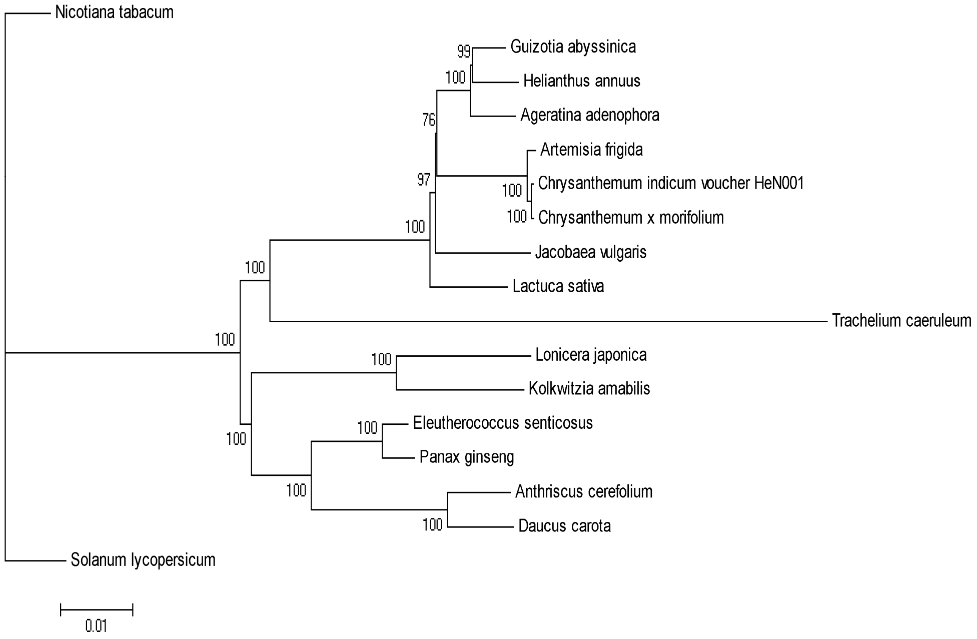

Figure S1. Evolutionary relationships of taxa. The evolutionary history was inferred using the Neighbor-Joining method [1]. The optimal tree with the sum of branch length = 0.33269139 is shown. The tree is drawn to scale, with branch lengths in the same units as those of the evolutionary distances used to infer the phylogenetic tree. The evolutionary distances were computed using the Poisson correction method [2] and are in the units of the number of amino acid substitutions per site. The analysis involved 17 amino acid sequences. All positions containing gaps and missing data were eliminated. There were a total of 51973 positions in the final dataset. Evolutionary analyses were conducted in MEGA5.

Table S1. List of genes found in *L. japonica* chloroplast genome (total 113 genes).

Category for Genes	Group of Genes	Name of Genes
Self replication	rRNA genes	*rrn16 rrn23 rrn4.5 rrn5*
	tRNA genes	*trnA-UGC trnC-GCA trnD-GUC trnE-UUC trnF-GAA trnfM-CAU trnG-GCC trnG-UCC trnH-GUG trnI-CAU trnI-GAU trnK-UUU trnL-CAA trnL-UAA trnL-UAG trnM-CAU trnN-GUU trnP-UGG trnQ-UUG trnR-ACG trnR-UCU trnS-GCU trnS-GGA trnS-UGA trnT-GGU trnT-UGU trnV-GAC trnV-UAC trnW-CCA trnY-GUA*
	Small subunit of ribosome	*rps2 rps3 rps4 rps7rps8 rps11 rps12 rps14 rps15 rps16 rps18 rps19*

	Large subunit of ribosome	*rpl2 rpl14 rpl16 rpl20 rpl22 rpl23 rpl32 rpl33 rpl36*
	DNA dependent RNA polymerase	*rpoA rpoB rpoC1 rpoC2*
	Translational initiation factor	*infA*
Genes for photosynthesis	Subunits of photosystem I	*psaA psaB psaC psaI psaJ*
	Subunits of photosystem II	*psbA psbB psbC psbD psbE psbF psbH psbI psbJ psbK psbL psbM psbN psbT psbZ*
	Subunits of cytochrome	*petA petB petD petG petL petN*
	Subunits of ATP synthase	*atpA atpB atpE atpF atpH atpI*
	ATP-dependent protease subunit p gene	*clpP*
	Large subunit of Rubisco	*rbcL*
	Subunits of NADH dehydrogenase	*ndhA ndhB ndhC ndhD ndhE ndhF ndhG ndhH ndhI ndhJ ndhK*
Other genes	Maturase	*matK*
	Envelop membrane protein	*cemA*
	Subunit of Acetyl-CoA-Carboxylase	*accD*
	c-type cytochrome synthesis gene	*ccsA*
	Genes of unknown function conserved open reading frames	*ycf1 ycf2 ycf3 ycf4*

Table S2. Size comparison of *L. japonica* chloroplast genomic regions with three other chloroplast genomes.

**Species**	**Length (bp)**				**Protein-Coding Genes**	**Duplicated Genes**	**Genes with Introns**
	**Genome**	**LSC**	**SSC**	**IR**			
Lonicera japonica	155,078	88,858	18,672	23,774	79	16	16
Daucus carota	155,911	84,242	17,567	27,051	81	21	18
Eleutherococcus senticosus	156,768	86,755	18,153	25,930	80	17	18
Panax ginseng	156,318	86,106	18,070	26,071	75	5	18

Table S3. Primers used for assembly validation.

**Primer**	**Sequence (5′>3′)**		**Amplicon Size (bp)**
LSC_IRb	F	TGCCCTGCGGTAATGATT	612
	R	TGCCAATAGGACCCTCCAA	
IRb_SSC	F	AACGCCTGAGAAGGACACT	405
	R	GGCAAGAAGGGTATTATCCA	
SSC_IRa	F	TCCATAATAGAGCCCGACC	678
	R	AACGCCTGAGAAGGACACT	
IRa_LSC	F	TGGCTCTGTATCAATGGA	717
	R	ATGCTCACAACTTCCCTC	

References

1. Saitou, N.; Nei, M. The neighbor-joining method: A new method for reconstructing phylogenetic trees. *Mol. Biol. Evol.* **1987**, 4, 406–425.
2. Zuckerkandl, E.; Pauling, L. Evolutionary divergence and convergence in proteins. In *Evolving Genes and Proteins*; Bryson, V., Vogel, H.J., Eds.; Academic Press: New York, NY, USA, 1965; pp. 97–166.
